# The laparoscopic hiatoplasty with antireflux surgery is a safe and effective procedure to repair giant hiatal hernia

**DOI:** 10.1186/1471-2482-14-1

**Published:** 2014-01-08

**Authors:** Luigi Marano, Michele Schettino, Raffaele Porfidia, Michele Grassia, Marianna Petrillo, Giuseppe Esposito, Bartolomeo Braccio, PierLuigi Gallo, Modestino Pezzella, Angelo Cosenza, Giuseppe Izzo, Natale Di Martino

**Affiliations:** 1 8th General and Gastrointestinal Surgery - Department of Internal Medicine, Surgical, Neurological Metabolic Disease and Geriatric Medicine, Second University of Naples, Piazza Miraglia 2, Naples 80138, Italy

**Keywords:** Giant hiatal hernia, Hiatoplasty, Antireflux surgery, Hiatal mesh reinforcement, Sac excision, Crural closure

## Abstract

**Background:**

Although minimally invasive repair of giant hiatal hernias is a very surgical challenge which requires advanced laparoscopic learning curve, several reports showed that is a safe and effective procedure, with lower morbidity than open approach. In the present study we show the outcomes of 13 patients who underwent a laparoscopic repair of giant hiatal hernia.

**Methods:**

A total of 13 patients underwent laparoscopic posterior hiatoplasty and Nissen fundoplication. Follow-up evaluation was done clinically at intervals of 3, 6 and 12 months after surgery using the Gastro-oesophageal Reflux Health-Related Quality of Life scale, a barium swallow study, an upper gastrointestinal endoscopy, an oesophageal manometry, a combined ambulatory 24-h multichannel impedance pH and bilirubin monitoring. Anatomic recurrence was defined as any evidence of gastric herniation above the diaphragmatic edge.

**Results:**

There were no intraoperative complications and no conversions to open technique. Symptomatic GORD-HQL outcomes demonstrated a statistical significant decrease of mean value equal to 3.2 compare to 37.4 of preoperative assessment (p < 0.0001). Combined 24-h multichannel impedance pH and bilirubin monitoring after 12 months did not show any evidence of pathological acid or non acid reflux.

**Conclusion:**

All patients were satisfied of procedure and no hernia recurrence was recorded in the study group, treated respecting several crucial surgical principles, e.g., complete sac excision, appropriate crural closure, also with direct hiatal defect where possible, and routine use of antireflux procedure.

## Background

Giant hiatal hernias is a rare condition characterized by more than 1/3 of stomach migration with or without other organs [1-3], representing 5-10% of all hiatal hernias [4]. These large hernias can occur asymptomatic or may cause a broad spectrum of signs and symptoms [5-9]. Typical symptoms are classically heartburn and regurgitation related to gastro-oesophageal reflux disease whereas atypical symptoms represent by vomiting and postprandial dysphagia owing to cavity reduction of the stomach herniated into posterior mediastinum, cough or dyspnea due to recurrent ab ingestis pneumonia and pulmonary compression as well as anemia subsequent to bleeding from gastric ulceration. Gastric strangulation, acute massive bleeding and perforation are rare but severe complications that may be found in emergency [5,8,9]. For this anatomical disruption the medical options are not adequate, therefore the surgical therapeutic strategy represents the only way to return to the past, to relieve the symptoms as well as to avoid serious complications [10]. In the past repair of giant hiatal hernia in open surgery (simple reduction, resection of the sac, gastropexy, and several types of fundoplication), transthoracically or transabdominally, is often considered the last chance for patients, above all for associated significant morbidities, mortalities, surgical pain and length of hospitalization [11-14]. In the last 10 years the widespread acceptance of minimally invasive surgery for upper abdominal surgery has changed the surgical approach to this hernias, preferring laparoscopic defect repair by means of simple reduction and posterior cruroplasty [15,16] or mesh reinforcement of posterior cruroplasty [6,17-22], followed by fundoplication procedure [23-25]. Although minimally invasive repair of giant hiatal hernias is a very surgical challenge which requires advanced laparoscopic learning curve, several reports showed that is a safe and effective procedure, with lower morbidity than open approach [26-29]. Nevertheless the laparoscopic management of giant hiatal hernia is still debated, while high mortality rate [30-32] and high recurrence rate [20,28,33,34] have been reported in recent evidences. In the present study we show the outcomes of 13 patients who underwent a laparoscopic repair of giant hiatal hernia.

## Methods

From January 2007 to December 2010 sixteen patients with giant hiatal hernia were treated at the 8^th^ General and Gastrointestinal Surgery Centre (Chief: Prof. Di Martino N) after treatment, for at least 3 months, with omeprazole at 80 mg daily. None of the patients had neoplastic disease. None had undergone earlier abdominal surgery and none had earlier gastrointestinal diseases. Three patients were excluded from the study needing open approach for absolute contraindications to laparoscopic approach: two patients were not able to tolerate general anesthesia and an open approach with combined spinal-segmental thoracic epidural anesthesia alone was indispensable; on the other hand the third patient was affected by chronic liver disease with refractory coagulopathy. A total of 13 patients underwent laparoscopic posterior hiatoplasty and Nissen fundoplication. In only two cases we added a mesh reinforcement of hiatoplasty. Before operation, all patients were interviewed about the presence of gastro-oesophageal reflux symptoms (heartburn, regurgitation and atypical symptoms) and the data were collected according to GORD-HRQL (gastro-oesophageal reflux disease - health related quality of life) [22] and SF-36 questionnaires [9-13]. After this, all patients underwent barium swallow, upper gastrointestinal endoscopy, combined thoracic and upper abdominal computerized tomography (CT) scan, stationary oesophageal manometry and simultaneous ambulatory 24-h multichannel impedance pH and bilirubin monitoring. Proton Pump Inhibitor (PPI), H2 receptor antagonist and prokinetic agents were discontinued 10 days before testing. Follow-up evaluation was done clinically at intervals of 3, 6 and 12 months from surgery. Symptoms of gastro-oesophageal reflux (heartburn, regurgitation), dysphagia, and other symptoms of hernia recurrence (abdominal pain, chest pain, gastric outlet obstruction, and evidence of gastro-oesophageal bleeding) were evaluated using the Gastro-Oesophageal Reflux Health-Related Quality of Life scale [34]. A barium swallow study was performed 3 and 12 months after surgery and an upper gastrointestinal endoscopy after 6 months. Oesophageal manometry and combined ambulatory 24-h multichannel impedance pH (MII-pH) and bilirubin monitoring after 3 and 12 months completed the postoperative follow-up evaluation. Anatomic recurrence was defined as any evidence of gastric herniation above the diaphragmatic edge. The study was approved by the ethics committee of Second University of Naples and conducted according to the ethical standards of the Helsinki declaration. Each patient gave informed written consent.

### Symptoms and quality of life

The GORD-HRQL questionnaire is an 11-item questionnaire designed to perform a practical, valuable and reliable evaluation of the severity of GORD-related symptoms and quality of life [22]. Each item is scored from 0 (best score) to 5 (worst score), with each score based on a descriptive anchor. The SF-36 questionnaire measures eight domains of HRQL using 36 items. These include physical functioning, physical role, bodily pain, emotional role, general health, social functioning and mental health. SF-36 scores for each health concept range from 0 to 100, with low scores representing poorer HRQL and a score of 100 representing the best possible HRQL.

### Barium swallow

In all the patients a standard oesophageal radiological examination after swallowing a bolus of contrast (Prontobario HD-Bracco, Milan, Italy) was obtained before surgery and at 3 and 12 months follow-up. On the basis of the literature we defined giant hiatal hernia as migration in thorax of more than 1/3 of stomach with or without other organs [4,35].

### Upper gastrointestinal endoscopy

Endoscopy was performed in all patients. The presence of oesophagitis was noted and graded according to the Los Angeles classification [36]. The presence and extent of hiatal hernia and Barrett oesophagus (defined by the presence of specialized intestinal metaplasia on biopsy taken above the gastroesophageal junction) were noted. Mucosal erythema, erosion or ulcerations of the gastric wall were considered signs of gastric inflammation.

### Combined thoracic and upper abdominal CT scan

All patients underwent combined thoracic and upper abdominal CT scan (Toshiba Aquilion, Toshiba Corporation, Tokyo, Japan) following an established protocol. Images were acquired in the supine position during a deep breath: the images were then reconstructed in axial mode by using an algorithm for soft tissue [33].

### Oesophageal Manometry

All subjects underwent stationary oesophageal manometry with an eight channel, multiple-lumen catheter (4 open tips at same level and oriented radially at 90° intervals and the other 4 extending proximally at 5 cm intervals) (Menfis Biomedica Inc. Bologna, Italy), perfused with a pneumo-hydraulic capillary infusion system (Menfis Biomedica Inc. Bologna, Italy). Resting pressure, total length and percentage of postdeglutitive relaxation, were the parameters used for the lower oesophageal sphincter (LOS) evaluation according to G.I.S.M.A.D. (Gruppo Italiano Studio Motilità Apparato Digerente) guidelines [37]. Oesophageal motor activity (amplitude and duration of waves, percentage of peristaltic and simultaneous postdeglutitive sequences) was evaluated with stationary pull-through after 20 dry swallows.

### Combined ambulatory 24-h multichannel impedance pH and bilirubin monitoring

After manometric evaluation, 24-h ambulatory combined oesophageal multichannel impedance pH monitoring and oesophago-gastric bilirubin monitoring was performed.

### Esophageal impedance/pH monitoring

The MII/pH probe (Sandhill Scientific Inc., Denver, CO, USA) was inserted transnasally and the distal pH probe was positioned 5 cm above the LES defined by earlier oesophageal manometry. Data from the impedance channels and the pH electrodes were transmitted at frequency of 50 Hz and stored on a portable data recorder (Sleuth; Sandhill Scientific Inc., Highlands Ranch, CO, USA). After 24-hours of recording, data was uploaded onto a personal computer and analyzed by using commercially available software system (Bioview Analysis; Sandhill Scientific Inc., Highlands Ranch, CO, USA).

### Combined pH and impedance data analysis

Acid reflux episodes were defined as a drop in pH to less than 4 for at least 5 seconds. The acid exposure percent time was calculated as the total time of the acid reflux episodes divided by the monitoring time. The DeMeester score was used to evaluate acid reflux. The acid exposure percent time was calculated as the total time of acid reflux episodes divided by the monitoring time. The bolus exposure percent time was defined as the sum of the bolus clearance time, of all individual reflux episodes, divided by the monitoring time. The bolus clearance time was defined as the time from a drop in impedance to 50% of the baseline value to recovery of 50% of the baseline value at the most distal impedance channel. The acid exposure percent time of the distal pH probe >4.0% or a DeMeester score >14.7 was defined as acid GORD. The bolus exposure percent time of more than 1.4% without acid GORD was diagnosed as nonacid GORD. The Symptom Index, defined as the percentage of symptoms associated with preceding reflux episodes occurring within 5 minutes time window obtained by dividing the total number of symptoms, was considered positive when ≥50%. The number of liquid and gas reflux episodes were also evaluated [38].

### Bilirubin monitoring

Two miniaturized fiberoptic probes (Medtronic Florence and Gad Medica s.n.c., Naples, Italy) were passed through the nose, positioning the proximal bilirubin sensor 5 cm above the upper border of the lower oesophageal sphincter (LOS), defined by earlier oesophageal manometry. The distal bilirubin sensors was located in the gastric body, 5 cm distal to the lower border of LOS. The patients were instructed to have three meals, avoiding dark-coloured foods, which interfere with Bilitec monitoring, according to a physiologic-standardized diet [39-41]. The data were registered and stored on portable digital recorders (Menfis Biomedical Inc. and Bilitec 2000) for 24 h and then the sensors were removed and the data downloaded into a personal computer for analysis.

### Bilirubin data analysis

Duodenogastric reflux (DGR) and duodenogastro-oesophageal (DGOR) bile reflux were defined as a bilirubin absorbance greater than 0.14, according to other authors and their validation studies [42]. The total, upright and supine percentage of oesophageal bilirubin exposure (normal values <7, <10.3, <1.8%, respectively) [43,44], were the variables used for the analysis of oesophageal bile reflux.

### Statistical analysis

Values are expressed as mean ± standard deviation. Continuous data were compared between each group using the one-way analysis of variance, paired *t* test, χ^2^ test or the Mann–Whitney U-test, when indicated. Prevalence data were compared between groups using Fisher’s exact test. p < 0.05 was considered to be statistically significant.

### Surgical procedure

**Figure 1:**
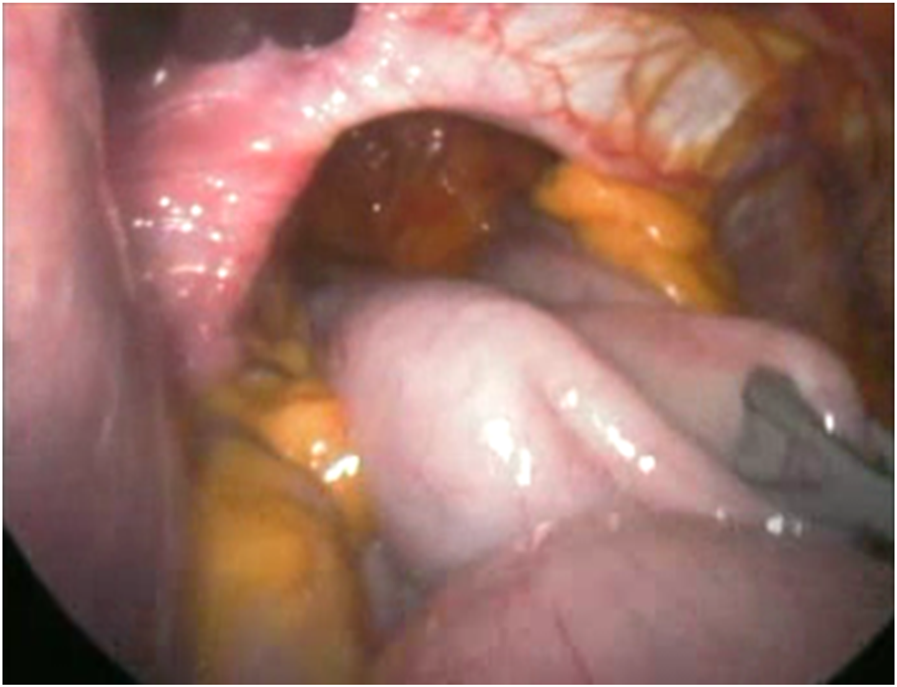
Laparoscopic view of a giant hiatal hernia with more than 50% of the stomach migrated in the chest throughout a large hiatal defect.

**Figure 2:**
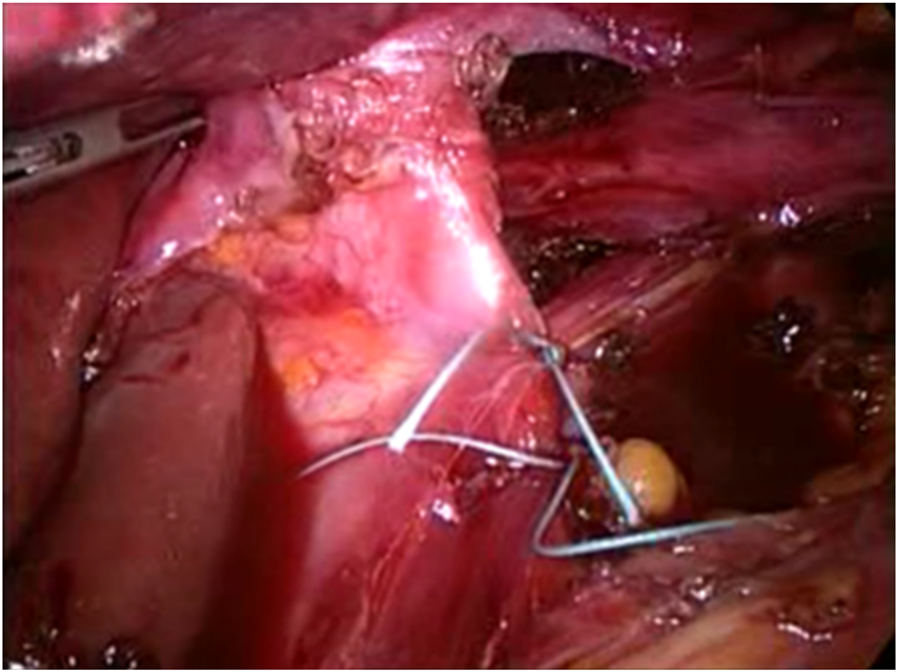
The hiatus is repaired with a series of interrupted sutures.

A nasogastric tube is routinely passed to decompress the stomach if it is found to be full of gas at laparoscopy. The patient is positioned in the lithotomy position (French position) and about 30° head up (reverse Trendelenburg). The videomonitor is placed at the patient’s eye level and in line with the operating surgeon who stands between the legs of the patient. The surgeon’s assistant stands at the patient’s left side. We generally use three 5-12-mm trocars and other two 5-mm trocars (Endopath Xcel™, Ethicon Endo Surgery, Blue Ash, OH, USA). Operating surgeon uses two atraumatic grasping instruments and Harmonic Scalpel (Harmonic ACE™, Ethicon Endo Surgery Europe, Norderstedt, Germany); furthermore it would be possible to use a 10-mm Ligasure™ device (Covidien, Boulder, CO, USA) but it is necessary the replacement of a 5-mm trocar with a 10-mm trocar. The assistant control a laparoscopic Babcock grasper, oesophageal retractor (Endoretract – Maxi™, Covidien Autosuture Mansfield, MA, USA) and a a 30° laparoscope. A 5-12-mm Hasson trocar is introduced 2–3 cm supraumbilically using an open insertion technique and pneumoperitoneum is established. The liver retractor is introduced via a 5-12-mm stab wound, which is placed as high as possible in the angle between the xiphoid and the apex of the left costal margin, to the left side of the falciform ligament. With this device, the left lobe of the liver is retracted upward and slightly to the right, to expose the hiatal defect. Three further ports are placed next: a 5-mm port immediately subcostal in the right midclavicular line, a 5-12-mm trocar at the cross between left midclavicular line and transverse umbilical line, and a 5-mm port in the anterior left axillary line approximately 3 to 4 cm below the costal margin. The size of the hernia and the contents of the hernia sac are first inspected (Figure 1). However, no attempt is made to reduce the contents of the hernia, as this is usually not feasible in very large hernias until the hernia sac has been fully dissected. Therefore the first step is to divide the lesser omentum to expose the right hiatal pillar. We start dissection of the sac at its neck by dividing the layers of the hernia sac, close to but 0.5–1 cm inside the hiatal rim. Our preference is to commence this dissection anterolaterally on the left side of the hiatus. The plane of dissection is then extended across the front of the hiatus toward the right pillar and posteriorly along the left pillar. Dissection alternates between the left and right sides of the hiatus, and the sac is gradually reduced into the abdomen. At this time, the hernia sac is dissected in the mediastinum using predominantly blunt dissection although the Harmonic scalpel is used if small blood vessels are encountered. This technique is mandatory to perform a retro-oesophageal window for the positioning of oesophageal retractor, pulling up the oesophagus and exposing the hiatus. It is important to undertake sufficient dissection to enable the posterior confluence of the hiatal pillars to be fully identified and for no hernia sac to lie anterior to this. In the process of posterior dissection, we routinely spare the posterior vagal nerve away from the esophagus. The hiatus is next repaired with sutures. The left and right pillars are approximated with two or more interrupted figures of 2/0 nonresorbable monofilament stiches, commencing posteriorly and working anteriorly in 5-mm steps, until the hiatus is reduced to a diameter of approximately 30 mm (Figure 2). If the repair appears to be under excessive tension, additional sutures can be placed in the anterior hiatus. If the crura were unable to be re-approximated without tension, or if the crura were attenuated or denuded of overlying peritoneum such that the ability to hold suture was compromised, mesh reinforcement with a mixed mesh (polypropylene + PTFE) Intramesh®T1 (Cousin Biotech, Wervicq-Sud, France) was performed. Large hiatal openings with intact peritoneal lining and well-developed mobile crura were closed primarily whenever possible, without routine mesh reinforcement. In 2 cases, with extremely thin and too stretched crura we changed to a mesh “tension-free” hiatoplasty. The hiatal defect was repaired with a mixed mesh fashioned in a U shape with a cradle for the esophagus, then fixed carefully to the edges of the diaphragmatic defect with six or more titanium helical tackes by means of ProTack™ 5 mm Fixation Device (Covidien Surgical, Mansfield, MA, USA). Once the hiatus is adequately narrowed, we add either a Nissen fundoplication to the procedure. We construct a loose 360° fundoplication using the anterior wall of the fundus of the stomach. In every case, the division of short gastric vessels was not necessary. During all the surgical procedures, the fundoplication were calibrated through endoscopy and manometry, by means of the same instruments used for patients’ preoperative evaluation. Particularly, the endoscope was inserted transorally at the beginning of the surgical procedure. The identification of the squamocolumnar junction, through the transillumination properties of the endoscope, was used to facilitate the identification of the stomach and the dissection of the lower esophagus. The intraoperative manometry was performed placing the catheter in the stomach by means of a guidewire. As concerns the endoscopic and manometric calibration of the esophageal wrap, the fundoplication was considered inadequate (too tight, misplaced or asymmetric) when a difficult transit of the endoscope through the wrap occurred, when the position of the wrap in relation to the squamocolumnar junction (SCJ) was not correct (less than 1 cm above the SCJ), the internal aspect of the wrap seemed irregular and interrupted on the retroversion views and when the neo-sphincter resting pressure exceeded 40 mmHg. According to the intraoperative endoscopy and manometry, whenever the fundoplication was not effectively calibrated, the surgeon refashioned it correctly. The fundoplication is not sutured to the diaphragm. Our standard approach to hiatal repair does not include reinforcement with mesh. Finally a naso-gastric tube is inserted and it will be removed during the first postoperative day. An abdominal drainage is placed near diaphragmatic hiatus. Oral food intake can start during second postoperative day. All patients were treated postoperatively with preventive omeprazole 40 mg daily therapy per os for one month.

## Results

A total of 13 patients, 5 males (38.5%) and 8 females (61.5%), with a median age of 67 years (range 37–71) were operated of laparoscopic posterior hiatoplasty and Nissen fundoplication. None of the patients had previously undergone surgery for gastro-oesophageal reflux disease or hiatal hernia.

### Preoperative findings

**Figure 3:**
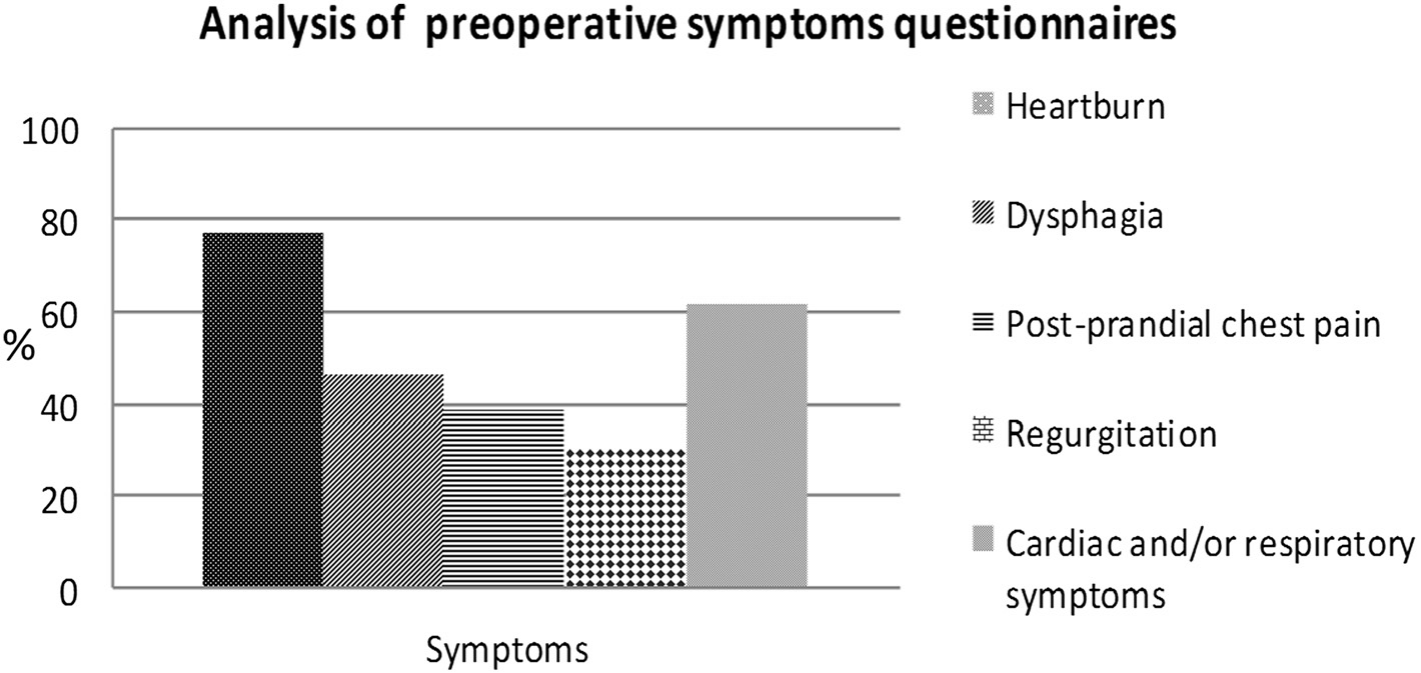
Analysis of preoperative symptoms questionnaires.

**Figure 4:**
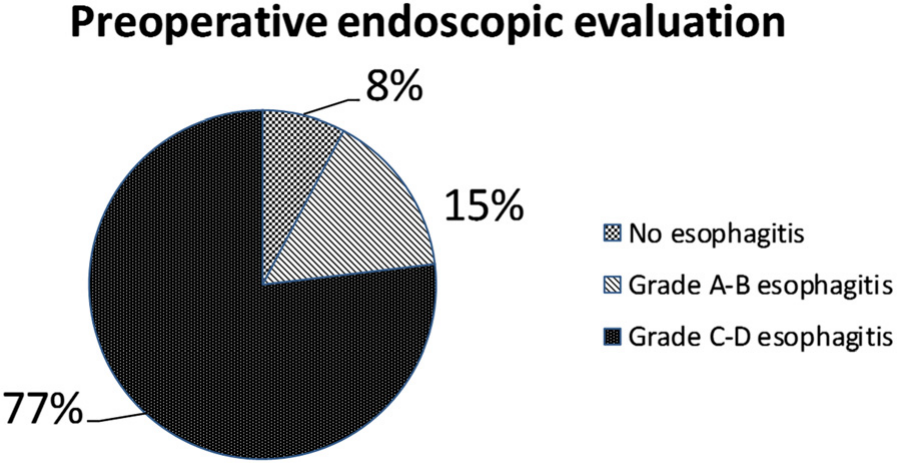
Preoperative endoscopic evaluation.

The analysis of symptoms questionnaires showed that 10 patients (77%) had heartburn, 8 patients (61.5%) had cardiac and/or respiratory symptoms, 6 patients (46.1%) had dysphagia, 5 patients (38.4%) had post-prandial chest pain and 4 patients (30.7%) had regurgitation (Figure 3). Preoperative GORD-HRQL questionnaire mean value was 37.4. At esophagogram all patients showed a giant hiatal hernia, with intrathoracic migration of more than 1/3 of stomach. Endoscopic evaluation revealed no esophagitis in 1 patient (7.7%), grade A-B oesophagitis in 2 patients (15.4%) and grade C-D esophagitis in 10 patients (76.9%) (Figure 4). Mean resting LOS pressure in population study was 9.3 ± 2.1 mmHg. 8 patients (61.5%) showed positive result by multichannel impedance pH monitoring data, with mean total percentage of oesophageal acid exposure (pH < 4) equal to 8.2 ± 0.8%. 5 subjects (38.4%) had the result that total bolus reflux percent time was over 1.4% (non acid reflux); moreover the Symptom Index was positive in 11 patients (84.6%). Concerning the refluxate characteristics, the mean of liquid reflux episodes was 28.5 ± 8.1 and the mean of gas reflux episodes was 14.7 ± 5. Abnormal oesophageal bilirubin exposure was found in 5 patients (38.4%), with mean total percentage of oesophageal bilirubin absorbance greater than 0.14 equal to 8.4 ± 0.5%. 2 of them (40%) showed combined abnormal gastric bilirubin exposure also, with mean total percentage of gastric bilirubin absorbance greater than 0.14 equal to 62.1 ± 6.2%.

### Surgery and early postoperative outcomes

There were no intraoperative complications and no conversions to open technique. The mean operative time was 128.3 ± 16.3 min for intervention without mesh and 156 ± 21.2 min for the mesh reinforcement technique. There was no mortality. Postoperatively, 2 patients (15.3%) had transient subcutaneous emphysema in the neck that resolved spontaneously in few hours; other two patients (15.3%) had pleural effusion adsorbed after a few days. No other postoperative complications were observed. Mean time to canalization was 1.4 ± 0.6 days. The mean hospital stay was 5 days (range, 4–7 days).

### Follow-up assessment

Follow-up evaluation was done clinically at intervals of 3, 6 and 12 months after surgery. Symptomatic outcomes according to the GORD-HRQL questionnaire were assessed at 12 months after surgery, demonstrating a statistical significant decrease of mean value equal to 3.2 compare to 37.4 of preoperative assessment (p < 0.0001). Esophageal swallow at 12 months after surgery did not find hernia recurrence, and upper endoscopic was normal in all patients after 6 months from intervention. Esophageal manometry 3 months after surgery showed a neo-high pressure zone mean value of 23.4 ± 2.1 mmHg, and 12 months after operation evidenced a mean value of 21.2 ± 1.6 mmHg, significantly higher than preoperative value (p = 0.045). No ineffective lower esophageal neo-sphincter was found. Combined 24-h multichannel impedance pH and bilirubin monitoring after 12 months did not show any evidence of pathological acid or non acid reflux. Interestingly, the postoperative percentage of Symptom Index was significantly lower than preoperative value (7.7% versus 84.6% respectively, p < 0.05). Furthermore, as concern the refluxate characteristics, the postoperative liquid reflux episodes mean of 21 ± 7.4 was significantly less frequent compared to 28.5 ± 8.1 of preoperative analysis, p < 0.0001, as well as the postoperative mean of gas reflux episodes (postoperative mean of 10.5 ± 5.1 versus preoperative mean of 14.7 ± 5, p < 0.05).

All patients completed the follow up assessment 12 months postoperatively and were satisfied with the outcome of procedure.

## Discussion

In the last years surgeons began to report experience with laparoscopic repair of large hiatus hernias, and this approach is now considered to be the technique of choice for the repair of large hiatus hernia [45]. However, controversy exists regarding four important issues of laparoscopic hernia repair: surgical approach (open or minimally invasive), the need of complete hernial sac excision, crural repair without or with mesh reinforcements and the exigency of an antireflux procedure addition. Primarily an open transthoracic approach, performing a left postero-lateral thoracotomy, was the technique of choice to giant hiatal resolution [28,46]. Anyhow it is well known that this access, when compared to most recent laparoscopic approaches, show an elevated mortality and morbidity, with longer convalescence [47]. In addition, during the early 90's, while laparoscopic techniques were developing, it was believed that the left open transthoracic approach would provide better access to the distal esophagus, allowing not only a best estimate of its length, but also the recourse to the Collis procedure in cases of short esophagus [28]. Progression to open transabdominal techniques for antireflux surgery in the 70's was also followed by the development of techniques for open abdominal hiatal hernia treatment [4]. All this is likely to be associated with less morbidity due to an abdominal rather than a chest incision, although there is not a high level of evidence to support these claims. It is, instead, well clear that both the thoracic and abdominal open approach are associated by high levels of postoperative pain, which is one of the most important trigger factors of perioperative morbidity. Many randomized controlled trials reported better short term operative and postoperative outcomes of laparoscopic Nissen fundoplication when compared to open technique [48]. Furthermore the quality of life and symptom score improvements related to laparoscopic correction of giant hiatal hernia are similar to those of open surgery, as reported in literature [3,28,49]. Our mortality rate (0%) is comparable to 0–3.7% mortality rate as reported by Hashemi, Low et al. [3,6,28,49]. In our population study there were no conversions to open technique and intraoperative complications. Postoperatively, only 2 patients (15.3%) had transient subcutaneous emphysema in the neck that resolved spontaneously in few hours and other two patients (15.3%) had pleural effusion adsorbed after a few days. Exactly the minimally invasive approach offers an excellent visualization of the hiatal region, far superior to that of laparotomy, and it is associated with low morbidity and mortality rates, a short hospital stay, and excellent patient compliance. From a technical point of view, during the phase of hernia reduction, the laparoscopic approach allows very precise identification of the anatomic structure (e.g., vagus nerves, parietal pleura, distal esophagus), and the dissection is facilitated by pneumoperitoneum [20,22,28]. One of the most crucial technical points in surgery for large hernias concerns the complete excision of hernia sac. The first procedures did not give proper importance to the dissection of the hernia sac, aiming to reduce the stomach completely laparoscopically and performing the preparation of the esophagus within hernia sac. This surgical strategy was not reliable, especially when stomach occupied more than 30-40% of hernia content; a conversion rate to open surgery of 40-50% was observed after first minimally invasive hernia reduction [41,50]. At the end of the 80’s Edye et al. [50] reported studies showing that when the laparoscopic approach focused initially on the dissection and reduction of the hernia sac from the mediastinum before performing esophageal mobilization, the conversion rate to open surgery was reduced to 10%. Recent experiences suggest that when the operation is performed by expert surgeons conversions to open surgery are quite uncommon [51], as in our series (no conversion rate was registered). The other debated issue is the need of mesh to reinforce the hiatoplasty. Surgeon who treats a giant hiatal hernia has to choose between the risk of hernia recurrence [52] and formidable complications due to mesh use [53]. Many scientific papers show that there is a significant decrease of 6-months hernia recurrence with hiatoplasty reinforcement by means of biomesh compared to only direct hiatus closure (9% versus 24%) [54]. We did not register any recurrence rate at 12 months follow up and our outcomes were in contrast with the high rates of radiographic recurrence published in some series of laparoscopic repair without mesh reinforcement [28,52,55-57]. We believe that it is possible to get satisfactory results even without using these devices, with the possibility of efficacy lost over long time, avoiding serious complications predicted by some authors in relation to the different type of meshes [58-62]: erosion or migration of the mesh into the esophagus or stomach, as well as complications due to severe mesh adhesions, infection, or the development of fibrotic strictures in the hiatal region. Although different series report complication rates from 1.3% [62] to 20% [58,63], the true rate of mesh-related complications is currently unknown [64], probably due to the lack of long-term follow-up studies. It should be noted that the use of meshes should limited, according to the surgeon personal experience, in cases characterized by extreme fragility of the pillars or excessive hiatus opening after direct closure, when the hiatoplasty seems inadequate. For these reasons, encouraged by the results presented by some authors [17-21], crural reinforcement is used in only two cases at our center, but without hesitation for problems with crural integrity or tension. In our opinion, although the study was conducted on a small number of patients with relatively short follow-up, to avoid the use of mesh are crucial complete dissection of sac and wide preparation of the oesophagus and diaphragmatic pillars to the maximum extent possible, to pull down the stomach and oesophagogastric junction in the abdomen for a length sufficient to prevent excessive pressure loading of the hiatal repair. This condition, therefore, is a prerequisite for a successful treatment. At this time, the decision to carry out a mesh cruroplasty rather than a direct closure after repairing a hiatal hernia is based on personal experience, and additional randomized studies on standardized use of reinforcement procedures are needed [10]. Another often discussed issue concerns the need for antireflux surgery in hiatal hernia repair. The extensive dissection required to obtain a quite long esophageal segment in the abdomen can make the gastroesophageal junction fail, resulting in postoperative reflux symptoms [10]. A number of authors [65,66] have reported an incidence of reflux symptoms in up to 47% of patients whose repair did not include any kind of fundoplication. It has been suggested that a fundoplication might reduce the recurrence rate by fixing the stomach in the abdomen, but this has not been demonstrated prospectively [10]. We performed an antireflux procedure in all the cases with a good control of reflux symptoms. Esophageal manometry 3 months after surgery showed a neo-high pressure zone mean value of 23.4 ± 2.1 mmHg, and 12 months after operation evidenced a mean value of 21.2 ± 1.6 mmHg, significantly higher than preoperative value (p < 0.05). Combined 24-h multichannel impedance pH and bilirubin monitoring showed at 12 months follow up no evidence of pathological acid or non acid reflux (p < 0.05) compared to preoperative assessment. Moreover we documented a reduction of liquid and gas reflux episodes after surgery, as a probable consequence of total fundoplication hindrance on the transient lower oesophageal sphincter relaxations due to the compression on the lower oesophageal sphincter segment, complying the observations of Linke GR et al. carried out in patients with proven GORD or hiatal hernia treated with laparoscopic mesh-augmented hiatoplasty without fundoplication [67]. Interestingly the postoperative mean total percentage of oesophageal acid exposure (pH < 4) and the postoperative Symptom Index of our series were also comparable with Linke GR et al. pH-impedance analysis results after laparoscopic mesh-augmented hiatoplasty without fundoplication [67].

## Conclusion

All patients were satisfied of procedure and no hernia recurrence was recorded in the study group, treated respecting several crucial surgical principles, e.g., complete sac excision, appropriate crural closure, also with direct hiatal defect where possible, and routine use of antireflux procedure.

## Competing interests

Authors have no competing interests or financial ties to disclose.

## Authors’ contributions

All authors approved the final manuscript. NDM and LM *concepted and designed the study*; LM, BB, MS, RP *acquired the data*; AC, LM, GI, MP *analyzed and interpreted the data*; LM and MS *drafted the manuscript*; NDM, LM, GI *performed a critical revision of the manuscript for important intellectual content*; MG, GE, AC *performed a statistical analysis*; NDM and LM *supervised the study*.

## Pre-publication history

The pre-publication history for this paper can be accessed here:

http://www.biomedcentral.com/1471-2482/14/1/prepub
